# A Low Cost Compact Measurement System Constructed Using a Smart Electrochemical Sensor for the Real-Time Discrimination of Fruit Ripening

**DOI:** 10.3390/s16040501

**Published:** 2016-04-08

**Authors:** Liuzheng Ma, Ling Wang, Ruipeng Chen, Keke Chang, Shun Wang, Xinran Hu, Xiaohui Sun, Zhaohui Lu, Haifeng Sun, Qingqian Guo, Min Jiang, Jiandong Hu

**Affiliations:** 1 Department of Electrical Engineering, Henan Agricultural University, Zhengzhou 450002, China; mlz0124@126.com (L.M.); wangling0351@126.com (L.W.); chen_ruipeng@yeah.net (R.C.); changkeke927@163.com (K.C.); wangshun6518@163.com (S.W.); xiaohuosun89@163.com (X.S.); zhaohuilulu@163.com (Z.L.); haifengdreams@sina.cn (H.S.); guo_qingqian@163.com (Q.G.); 2 State Key Laboratory of Wheat and Maize Crop Science, Zhengzhou 450002, China; 3 School of Human Nutrition and Dietetics, McGill University, Macdonald Campus, 21, 111 Lakeshore Road, Ste Anne de Bellevue, QC H9X 3V9, Canada; xinran.hu@mail.mcgill.ca; 4 College of Life Sciences, Henan Agricultural University, Zhengzhou 450002, China; jm_hu@163.com

**Keywords:** ethylene gas, micropump, electrochemical sensor, fruit ripening, non-invasive

## Abstract

Ethylene as an indicator for evaluating fruit ripening can be measured by very sensitive electrochemical gas sensors based on a high-resolution current produced by a bias potential applied to the electrodes. For this purpose, a measurement system for monitoring ethylene gas concentrations to evaluate fruit ripening by using the electrochemical ethylene sensor was successfully developed. Before the electrochemical ethylene sensor was used to measure the ethylene gas concentrations released from fruits, a calibration curve was established by the standard ethylene gases at concentrations of 2.99 ppm, 4.99 ppm, 8.01 ppm and 10 ppm, respectively, with a flow rate of 0.4 L·min^−1^. From the calibration curve, the linear relationship between the responses and concentrations of ethylene gas was obtained in the range of 0–10 ppm with the correlation coefficient R^2^ of 0.9976. The micropump and a novel signal conditioning circuit were implemented in this measurement, resulting in a rapid response in detecting ethylene concentrations down to 0.1 ppm in air and in under 50 s. In this experiment, three kinds of fruits—apples, pears and kiwifruits—were studied at a low concentration (under 0.8 ppm) of trace ethylene content in the air exhaled by fruits. The experimental results showed that a low cost, compact measurement system constructed by using an electrochemical ethylene sensor has a high sensitivity of 0.3907 V·ppm^−1^ with a theoretical detection limit of 0.413 ppm, and is non-invasive and highly portable.

## 1. Introduction

There has been increased scientific interest to gradually establish a well-functioning, high quality fruit evaluation system for food safety. Maturity at harvest is the most important factor that determines storage-life and fruit quality. Correspondingly, maturity signifies that fruits are acceptable for purchase and are optimal for consumption. However, immature fruits are more subject to shriveling and mechanical damage [1,2]. For example, overripe fruits are likely to become soft and mealy with an insipid flavor soon after harvest. Generally speaking, some features including skin color, shape, size, flavor, smell, firmness and echoes produced by gently tapping the surface of fruits are the most currently used maturity indices for evaluating fruit ripening. However, such assessments are too subjective in order to reliably identify fruit ripening levels [2]. The biological, physiological, mechanical and physical characteristics of fruits are changing [2,3,4]. Furthermore, some factors stemming from inadequate handling during harvest and post-harvest in transportation, packaging and storage must be comprehensively considered to preserve the quality of fruits [4,5]. Chemically, sugar concentration, acidity, and starch contents are the customary indices used to evaluate fruit ripening. These invasive methods provide the information needed to accurately identify fruit ripening levels [6]. However, it is time consuming to determine the correct values of parameters to adequately evaluate fruit ripening. In recent years, visible-near-infrared (vis-NIR) spectroscopy has been shown to be a promising, nondestructive method to evaluate fruit ripening, since it provides reliable information on the internal characteristics of various fruit species [7,8,9]. However, this approach requires exceedingly complex models in the data processing stage to obtain precise results for evaluating fruit ripening. A novel, noninvasive combination optical method based on reflectance and fluorescence spectroscopies together with the gas in the scattering media absorption spectroscopy (GASMAS) technique was developed to study the ripening period of tropical fruits [10]. However, ethylene, a gaseous organic compound in fruits and widely known to be involved in the ripening process, is not considered in portable instruments. Fruits such as apple, pear, quince, persimmon, apricot, nectarine, peach, plum, kiwifruit, banana and mango *etc.* produce large amounts of ethylene gas in association with their ripening [11,12,13]. To monitor and control the ethylene emission in growth chambers, greenhouses and storage facilities are of paramount importance to optimize fruit freshness because ethylene can stimulate fruit ripening even at levels of tens of nL·L^−1^ [14]. Several groups have reported on the detection of ethylene in fruits. Gas chromatography (GC) is a common type of chromatography for separation and analysis of volatile compounds in many research and industrial laboratories [15]. Although many applications of GC have been used to measure ethylene from fruit, it is a time consuming process and is not easily reproduced as the sampled amount could differ slightly [16,17,18,19]. An olefin detector based on the metal-organic framework was obtained to measure the ethylene gas by quenching fluorescence [20]. An electrochemical ethylene sensor constructed by a C60/Zeolite semiconductor electrode was investigated to monitor the ethylene gas in a wide concentration range [21]. A low-cost gas sensor produced by the graphite line-patterning technique was used to characterize banana ripening by examining the distinct pattern of signals [22]. The use of an electronic nose for non-destructively monitoring the fruit ripening process is also presented. Based on a tin oxide chemical sensor array and neural network-based pattern recognition techniques, the photoacoustic technique for trace ethylene gas detection has proved to be a sensitive and reliable method [23,24,25,26,27]. A measurement set-up constructed by using an electrochemical sensor was able to detect ethylene gas down to six parts per trillion (ppt) with a time response of 2 s by optimizing the photoacoustic cell [27]. The sensor that employs a thin layer of ionic liquid as electrolyte was developed to detect the concentrations of the ethylene [28]. A sensing mechanism with high sensitivity to the resistance of single-walled carbon nanotubes in order to suppress their electronic surroundings can detect sub-ppm concentrations of ethylene [29]. Despite these results, smart electrochemical sensors have not yet been used for monitoring the ethylene gas released from fruits. For fruit maturity detected by a compact measurement system, the ethylene gas measurements will have much significance. In the present article, we demonstrate a low cost, portable, noninvasive measurement technique to evaluate fruit maturity. A smart electrochemical ethylene sensor was used in combination with a microcontroller and a micropump to monitor the changes of ethylene gas concentration during fruit growth. A kinetic model of the responses from the smart ethylene electrochemical sensor was established, which was a real-time quantitative evaluation approach to virtually investigate ethylene emission from fruits. The levels of fruit maturities can be discriminated with 85% (Overripe), 60% (Ripe), and 30% (Underripe), respectively, in accordance with ethylene concentrations released from fruits.

## 2. Materials and Methods

### 2.1. Materials

The smart electrochemical ethylene sensor with the detection ranges of 0–10 ppm was customized from Shenzhen Shengkai Technology Co. Ltd (Shenzhen, China). A very good linear relationship with a nonlinearity of 0.2% between the response voltages and the ethylene concentrations should be achieved. The response voltages of 0–5 V correspond to the ethylene concentrations in the range of 0–10 ppm, respectively. The response voltage is zero from the electrochemical ethylene sensor by flowing nitrogen gas through the gas chamber at the flow rate of 0.4 L·min^−1^. A gas chamber was constructed by one circular glass plates, with 5 mm thickness and 20 mm diameter, and the inlet and outlet ports with 6 mm diameter. Typically, this electrochemical ethylene sensor offers a 0.1 ppm resolution. The temperature compensation circuit was embedded into this electrochemical sensor. A microcontroller was integrated with the electrochemical sensor to realize data communication between the electrochemical sensor and the upper microcontroller, either in voltage mode or digital mode. The standard ethylene gases at the concentrations of 2.99 ppm, 4.99 ppm, 8.01 ppm and 10 ppm, respectively, with an accuracy of 0.01 ppm were prepared by the Measurement Engineering Technology Research Center of Henan Province (Zhengzhou, China) in accordance with the Chinese National Standard (GB/T5274-2008, Gas analysis-Preparation of calibration gas mixtures-Gravimetric method). Correspondingly, the certified reference material of pure ethylene gas GBW(E)060036 was obtained from the National Institute of Metrology, China (Beijing, China). It is used to establish the standard curve to evaluate the performance of this electrochemical sensor for the measurement of ethylene gases. Three kinds of fruits such as apple, pear and kiwifruit for evaluating fruit ripening were purchased from the local supermarket. Before this experiment, the fruits were washed and dried by air.

### 2.2. Principle of the Smart Electrochemical Ethylene Sensor

**Figure 1:**
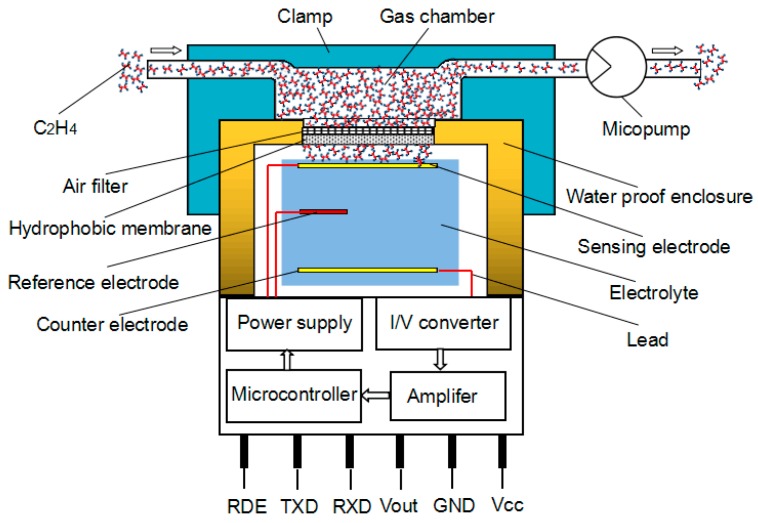
Schematic diagram of the smart electrochemical ethylene sensor.

Electrochemical sensors have the advantages of small size, simple operation and portability, and can be conveniently used for on-site monitoring. Therefore, electrochemical sensors play a very important role in the detection of various types of gas. The electrochemical ethylene sensor is operated by reaction with ethylene. It can transform the concentration of the ethylene gas into an electrical current signal. For smart electrochemical ethylene sensors, a small current is generated, which is linearly proportional to the ethylene gas concentration due to a microcontroller embedded into it. The current-potential relationship was obtained from the electrochemical reaction where equilibrium is not established. In this experimental setup, the smart electrochemical ethylene gas sensor consists of a diffusion barrier, a working-electrode, a counter-electrode, a reference electrode and an electrolyte, as well as a layer of phosphoric acid (see Figure 1). The phosphoric acid electrolyte solution used in this electrochemical sensor possesses an electrochemical stability and is not easily crystallized. Furthermore, the conductivity-viscosity product of the phosphoric acid electrolyte was found to decrease exponentially with increasing temperature [30]. Therefore, an extended-life electrochemical sensor using phosphoric acid can be achieved. The working electrode is commonly fabricated by fixing a precious gold anode with a high surface area onto a porous hydrophobic membrane, while counter electrode and reference electrode are thin-film platinum electrodes, respectively. The hydrophobic membrane, also called gas permeable membrane is a dip-coated polystyrene. The external dimensions of the electrochemical ethylene sensor are 31 mm long and about 33.5 mm in diameter. However, the planar-type counter- electrode is 10.4 times larger than the area of the working electrode for obtaining high sensitivity. When a voltage is sent to the gold electrodes, ethylene is catalytically oxidized at the surface of the gold electrode after it is diffused into the electrochemical reaction cell through the barrier by the micropump. The current is quantitatively related to the rate of the electrolytic process at the working electrode whose potential is usually kept constant by using the so-called reference electrode. The reference electrode is used to eliminate interference from side reactions with the counter electrode. In addition, it allows the working-electrode potential to be biased. Biasing is one method of controlling sensitivity to the ethylene gas. In the presence of ethylene gas, the response value peak appeared around 0.7 V (peak voltage at the bias potential ranged from 0.5 to 1.11 V), which was obtained from the power supply embedded into the smart electrochemical ethylene sensor. Ethylene is oxidized at the working electrode:

(1) $$C_{2}H_{4}+4H_{2}O\to 2CO_{2}+12H^{+}+12e^{-}$$

The counter electrode acts to balance out the reaction at the sensing electrode by reducing oxygen present in the air to water:

(2) $$O_{2}+4H^{+}+4e^{-}\to 2H_{2}O$$

The results obtained from chemical reactions were satisfied by Butler-Volmer equation, in which the limiting current density can be expressed by the following Equation (3).

(3) $$I_{\lim}=\frac{n\cdot F\cdot D\cdot C_{C_{2}H_{4}}}{\delta }$$

where, *I_lim_* is the limiting current density; *n* is the number of transferred electrons; *F* is the Faraday constant; *D* is the diffusion coefficient of ethylene gas C_2_H_4_; C_C_2_H_4__ is the concentration of ethylene gas C_2_H_4_ in the bulk of the electrolyte; *δ* is the diffusion layer thickness, which depends on the sensor's geometry. Therefore, if the working electrode behaves ideally, the limiting current density would be proportional to the concentration of the ethylene gas C_2_H_4_. These electrochemical sensors are typically operated at a high over potential; for example, the current is independent on the applied potential and scales linearly with ethylene concentrations, as indicated in Equation (3). Equation (3) provides a current–concentration relationship for an electrochemical ethylene gas reaction. The current increases exponentially with the gas concentrations. At a high overvoltage, diffusion limits the current and approaches a steady state if the electrochemical sensor is designed correctly. Experimental results show that the current is proportional to the concentration of the ethylene gas. The sensor had a detection limit down to 0.1 ppm of ethylene in gas concentrations ranging from 0 to 10 ppm. Moreover, the baseline draft obtained from the electrochemical sensor suffered from the instable flow rate of ethylene gas controlled by the micropump.

## 3. Monitoring of the Ethylene Gas with a Smart Electrochemical Sensor

**Figure 2:**
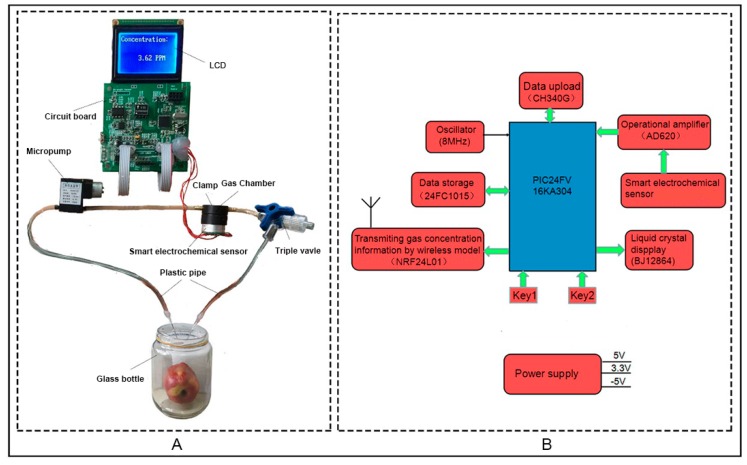
Experimental setup for the ethylene measurement using an electrochemical sensor: schematic diagram and photograph of setup. (**A**) The measurement apparatus incorporates all of the components (**B**) The circuit schematic structure of the measurement system for monitoring the ethylene gas in real time.

Ethylene is an invisible, colorless and odorless gas, which has no known hazardous effect on human beings at the concentrations encountered within fruit. The relatively small and simple ethylene molecule consists of two carbon atoms associated with four hydrogen atoms, and the molecular weight of ethylene is 28.05  g·mol^−1^ [31]. As we all know, in the process of fruit ripening, ethylene gas is gradually released according to fruit weight and storage time. Therefore, the determination of the ethylene concentration released from fruits can be a convenient method for evaluating the maturity of fruits. A detailed description of the measurement system using a smart electrochemical ethylene sensor system is shown in Figure 2. The electrochemical sensor-based ethylene measurement system consists of a micropump, a microcontroller, an electrochemical ethylene sensor and a triple valve. Traces of ethylene released from fruits can react with the electrolyte inside the electrochemical sensor under the constant working voltage applied between a working electrode and a reference electrode. By controlling the working voltage, a small current is generated in the counter electrode, which can be converted into voltage with a current to voltage (I-V) conversion circuit embedded into the electrochemical sensor. Accordingly, the ethylene concentration is calculated by the voltage from the electrochemical sensor. The electrochemical sensor-based measurement system allows monitoring the ethylene emission in a continuous flow system down to 0.1 ppm.

As ethylene diffuses freely, considerable residual ethylene levels may also remain in the gas chamber where fruit had previously been stored, depending on the amount, stage of ripening, type of fruit previously stored there and storage time. From Figure 2A, the gas chamber was constructed by one cylindrical plastic barrel, with 5 mm thickness and 20 mm diameter and two plastic pipes with 6 mm diameter. One compression ring, bolted together, was used to hold the chamber window together. The glass bottler was polished by hand to store the fruits. Two 2 mm holes were made in the center of the top plastic film, into which two plastic pipes were glued. The two plastic pipes were separated by the spacer of 25 mm width symmetrically placed around the circumference.

A circuit designed for the portable real-time measuring instrument is shown in Figure 2B, which includes a power supply circuit module, a wireless transmission module for transmitting I-V conversion signals, a microcontroller module for signal processing and an acquisition module, a data storage module, a data upload module and a display module. Microcontroller PIC24FV16KA304 is selected, which has a high performance CPU with modified Harvard architecture and the running speed of 16 MIPS. A 12-bit high precision A/D converter is embedded into this microcontroller to convert the analog signal to digital signal. The wireless transmitting module, mainly including chip NRF24L01, is utilized to communicate with other wireless transmitting modules, operating at the Industrial Scientific Medical (ISM) frequency band. Due to the output voltage from the electrochemical sensor in millivolt, the voltage signals should be amplified by instrumentation amplifier AD620 before heading to microcontroller PIC24FV16KA304. Data storage chip 24FC1015, an Electrically Erasable Programmable ROM with the maximum storage space of 1024 Kb at the operating voltage range of 1.7~5.5 V, was utilized to store the measurement results. The data upload module, constructed by the chip CH340G achieves the function of upgrading the serial port to the USB bus directly. The display module consisted of the chip of BJ12864F is interfaced with the microcontroller PIC24FV16KA304 to display the measurement results in 128 columns × 64 lines.

## 4. Measurements and Results

### 4.1. Establishment of the Standard Curve for the Quantification of the Ethylene Gas Concentrations

**Figure 3:**
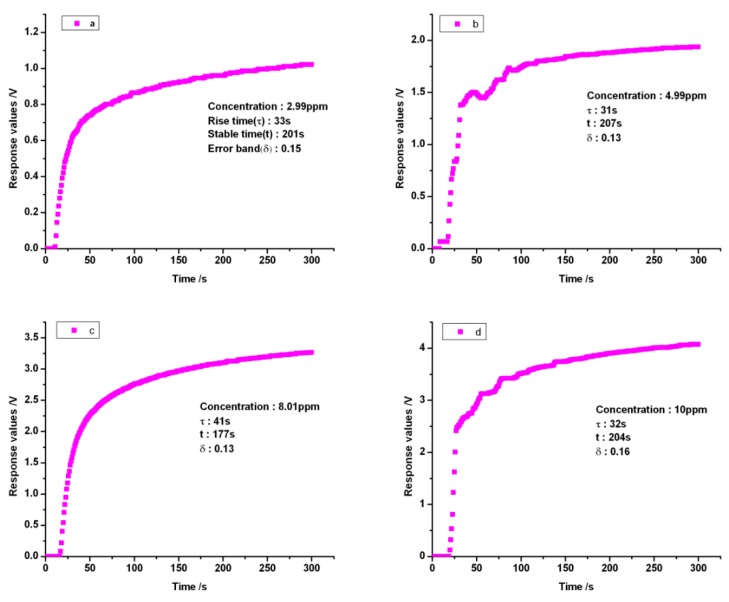
The time-response characteristics of the measurement system.

**Figure 4:**
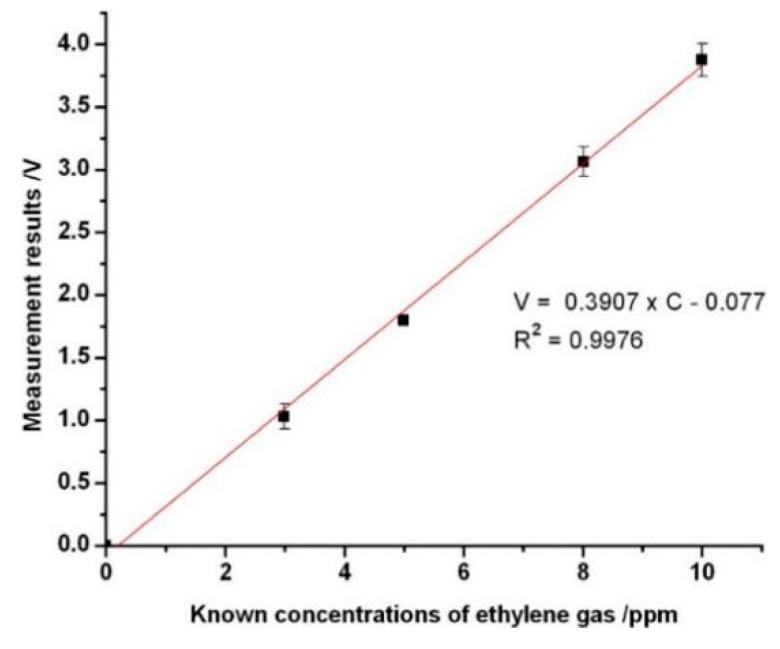
Establishment of the fitting curve for the quantification of the ethylene gas concentrations.

In this measurement system, ethylene gas dissolves in the electrolyte that covers the working, reference and counter electrodes. Owing to this oxidization, a small current is generated. This current is measured to determine the ethylene gas concentration in ppm, which was performed under dry conditions at room temperature. From Figure 2B, the real-time detection mode in the measurement software was launched by moving the corresponding cursor after this measurement system was powered on. The ethylene gas concentrations were monitored by the electrochemical sensor under the sampling rate of 20 Hz. This sensor was able to detect ethylene gas concentrations ranging from 0 to 10 ppm. The flow rate of the ethylene gas had a constant value of 0.4 L·min^−1^, which was controlled by the micropump during the measurement processing. Calibration is done with the known concentration ethylene gases. Concentrations of 2.99 ppm, 4.99 ppm, 8.01 ppm and 10 ppm with the highest accuracy of 0.01 ppm, respectively, were used for the calibration. Baseline measurements were collected from the gas chamber with room air flowing through, although electrochemical sensors are not affected directly by humidity. However, the dramatic changes in the relative humidity can change the water content in the electrolyte affecting the electrochemical reaction. Certainly, this process occurs very slowly and depends upon the temperature, the electrolyte, and the hydrophobic membrane. The effective values were the differences between the measurement values from the ethylene gas in the electrolyte cell and the baseline from air. The flow rate released from the gas bottle was adjusted by the gas valve to achieve an adequate value (0.4 L·min^−1^) corresponding to the pressure from the micropump. In the gas chamber, ethylene molecules go through the gas filter and the porous hydrophobic membrane to reach the working electrode of the electrochemical sensor after ethylene gas diffusion. The experimental results showed that the uneven flow rate of the ethylene from the micropump can fluctuate the electrochemical reaction relating to the response values from the electrochemical sensors. From Figure 3, it can be seen that, sometimes, the response values have risen sharply most likely from the fluctuation of flow rate due to the varied pressures existing in the micropump. The current signals from the electrochemical sensor were converted and amplified to be processed by the microcontroller PIC24FV16KA304. The measurement results indicated with maturity levels or concentrations were promptly displayed on the liquid-crystal display (LCD) screen. The output signal voltages ranged from 0 to 5 V, corresponding to the measurement ranges of 0–10 ppm obtained by the I-V converter and the operational amplifier in the smart electrochemical ethylene sensor. The response curves from four different ethylene gas concentrations of 2.99 ppm, 4.99 ppm, 8.01 ppm and 10 ppm shown in Figure 3 as a, b, c and d, respectively. From Figure 3, it can be seen that the response voltages that give rise to the concentrations of the ethylene gases increased rapidly. The shapes of the chronoamperometric transients well agree with the unit step response function (see Figure 3) due to the time period needed for the electrochemical reaction around the electrodes. After the time period of three or five time constants (τ), the stable state was reached finally. The response curves display the amount of jitter at the rising phase, which stem from the uneven pressures existing in the pipe from the micropump. For the unit step response function, the rate at which the response approaches the final value is determined by the time constant τ. When t = τ, the output has reached 63.2% of its final value. From each of the response curves (Figure 3), clearly, response values change from baseline to a relatively stable value within an error band of 7.14% in 300 s. The time constants were calculated from Figure 3a–d as 33 s, 31 s, 41 s and 32 s, respectively. Physically, the largest one of 41 s is the time constant with respect to the electrochemical ethylene gas sensor. The regression line was established by using the different ethylene concentrations (see Figure 4). From the regression line, the optimized relationship between the ethylene gas concentrations and response values was obtained with the correlation coefficient of 0.9976. The theoretical detection limit was calculated to be 0.413 from the formula 3σ/k, where σ is the residual standard deviation of the linear regression and k is the slope of the regression line.

### 4.2. Measurement of the Ethylene Concentrations Released from the Fruits

**Figure 5:**
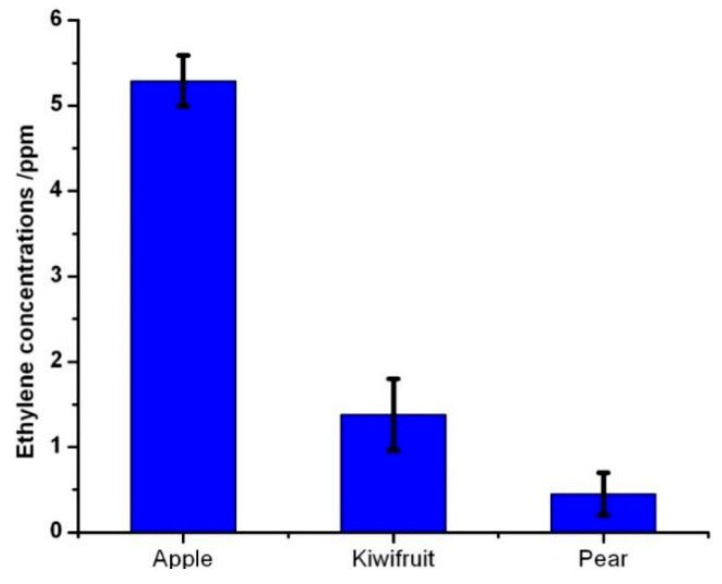
Measurements of ethylene gas concentration released from individual fruits including apple, kiwifruit and pear by using the electrochemical sensor for less than 1 min.

The glass bottles and three kinds of fruits—apples, pears, and kiwifruits—were bought from the local market. All of them were cleaned with tap water and dried naturally. The fruits were organized into three groups according to species. The three kinds of fruits were fitted into the sealed glass bottle at room temperature for 20 min, respectively. One end of the micropump is connected to the electrochemical sensor, while the other end is connected to the plastic pipe to form a closed loop by inserting it into the glass bottle, ensuring the fluctuating air from the micropump has no influence on the measurements. The ethylene gas released from the fruits was pumped into the gas chamber over the smart electrochemical sensor. Afterwards, ethylene gas was diffused over the electrolyte to the working electrode of the electrochemical sensor. The electrochemical sensor should be regenerated by air flowing through the chamber to remove the residual ethylene gas for the next measurement. From Figure 5, climacteric fruits such as apple, pear and kiwifruit have a distinctive response peak during the ripening process, whereas the peak concentration can be conveniently measured with this measurement system. In this experiment, the measuring range of 0–10 ppm ethylene gas concentration with a reading accuracy of ±0.5% was obtained before the calibration curve was established and the median filter algorithm was used. The average ethylene gas concentrations were measured to be 5.29 ppm, 1.38 ppm and 0.45 ppm from the measured samples of apple, kiwifruit and pear, respectively. The reproducibility of the ethylene values was 38%, 58% and 5% in relative standard deviation (RSD) with three subsequent measurements of apples, kiwifruits and pears, respectively. In particular, after a number of ripe and unripe fruits were examined by using this ethylene gas measurement system, an evaluation model was established according to the concentrations of ethylene gas released from the fruits. When the concentrations of ethylene gas were reached more than 1 ppm, the ethylene gas accelerated the fruit ripening. For apples, the average concentration of the ethylene gas was obtained to provide the theoretical basis for distinguishing three stages of ripening. For the unripe fruit, the concentrations of ethylene gas obtained from this measurement system were less than 1 ppm, while concentrations of the ethylene gas ranging from 1 to 6 ppm were roughly employed to evaluate the ripe fruit. For the concentrations averaging over 6 ppm, the fruits were considered to be overripe.

### 4.3. Selectivity and Regeneration

**Figure 6:**
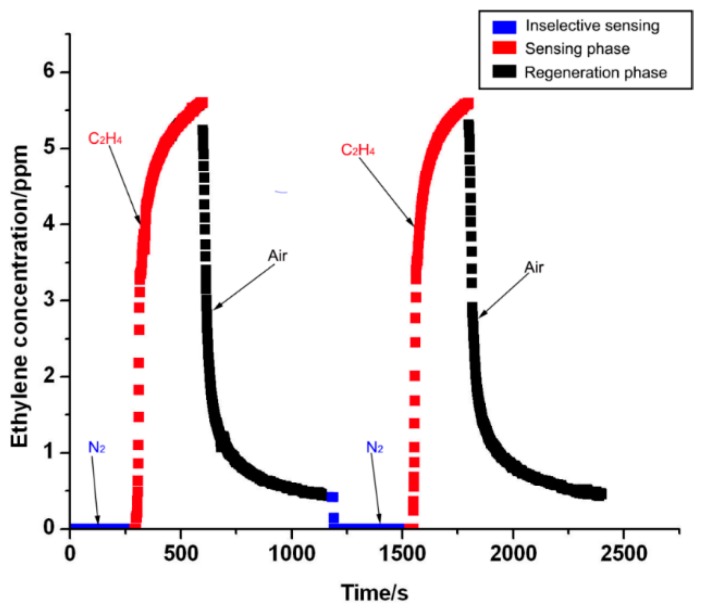
Response values from the selectivity experiment with nitrogen gas and the regeneration process of this electrochemical ethylene gas sensor.

This electrochemical sensor is very small and very sensitive to the ethylene gas. With a reading accuracy of ±0.5%, reproducibility indicated RSD of 38%, 58% and 5% for apples, kiwifruits and pears, respectively. The response time is calculated from the time that the response values were changed from the baseline to a relatively stable value within an error band of 7.14%. For this electrochemical ethylene gas sensor, an electrochemical reaction occurred between electrodes. Through the chamber of this measurement system, the gas with the ethylene molecules flowed over the electrolyte solution. As an electrochemical reaction occurred with the ethylene molecules, an increase in response values was observed. After the desired response time, gas without the ethylene molecules (usually the air gas) flowed over the chamber that determines the electrochemical reaction between the electrolytes and ethylene molecules. Here, as the air dissociates the electrochemical reaction, a decrease in response values was observed. Therefore, from Figure 6, it can be seen that a response time between 30 s and 2 min is well suited for the evaluation of fruit ripening.

The experimental results obtained from two repeated experiments are shown in Figure 6 with ethylene gas and air in turn. From Figure 6, it can be seen that the sensing response did not significantly change after the electrochemical ethylene sensor was reused more than two times. Here, the nitrogen gas was used (see the first phase in Figure 6) to evaluate the selectivity of this electrochemical sensor. The response value is approximately zero from the electrochemical sensor by flowing nitrogen gas through the gas chamber at the flow rate of 0.4 L·min^−1^. However, the air was used to dissociate the electrochemical reaction. Therefore, a decrease in response values can be easily observed in Figure 6. The regeneration time in relation to the response value was the baseline from the relatively stable value with an error value of 7.14%, which was obtained from the ethylene gas concentration. The regeneration was realized using other gas to remove ethylene molecules around the electrodes, such as air, because air is commonly diffused into ethylene gas. The regeneration time in relation to the response value was the baseline from the relatively stable value within an error value of 7.14%, which was obtained from a certain concentration of ethylene gas. From the experimental results shown in Figure 6, the regeneration time was calculated to be 350 s.

## 5. Conclusions

A low cost, noninvasive measurement system for the evaluation of fruit maturity was developed by using a smart electrochemical ethylene sensor in combination with a microcontroller and a micropump. The ball-park figure of this measurement system including the electrochemical ethylene gas sensor, the micropump and electronic devices would be around 300 US dollars. A detection limit of 0.413 ppm was obtained to quantify the ethylene gas concentration during the fruit ripening process. A kinetic model of the responses was established from the smart ethylene electrochemical sensor. The average ethylene gas concentrations were found to be 5.29 ppm, 1.38 ppm and 0.45 ppm for apple, kiwifruit and pear, respectively. The reproducibility of the ethylene values was 38%, 58%, 5% in RSD with three subsequent measurements of apples, kiwifruits and pears, respectively. The present results clearly show that it is possible to study the process of fruit ripening using nonintrusive electrochemical ethylene sensors. For fruit harvesting and marketing, it is very important to know the maturity and full ripening stages of the fruits. In order to obtain agreement with subject evaluations of fruit maturity, the thresholds for individual kinds of fruits have to be firmly established. For detecting trace ethylene gas concentration, the smart electrochemical sensor remains a powerful device, especially when used in combination with micropumps and microcontrollers. Smart electrochemical ethylene sensors seem to be very promising because of their high response values in detecting the slight changes in ethylene concentrations in fruits, their operation at room temperature and their great potential for compact instrumentation. The current measurement system for monitoring trace ethylene gas concentration is only suitable for the evaluation of a few species of fruits, such as apple, kiwifruit and pear, due to the limitation of established kinetic models. If a highly sensitive smart electrochemical ethylene sensor can be achieved, electrochemical sensor-based instruments would replace currently operated analytical instruments. In particular, for determining the ethylene released from fruits, the use of such an electrochemical sensor in combination with a micropump system has proven that it is an unbeatable approach in terms of sensitivity and time response in comparison with traditional methods.
